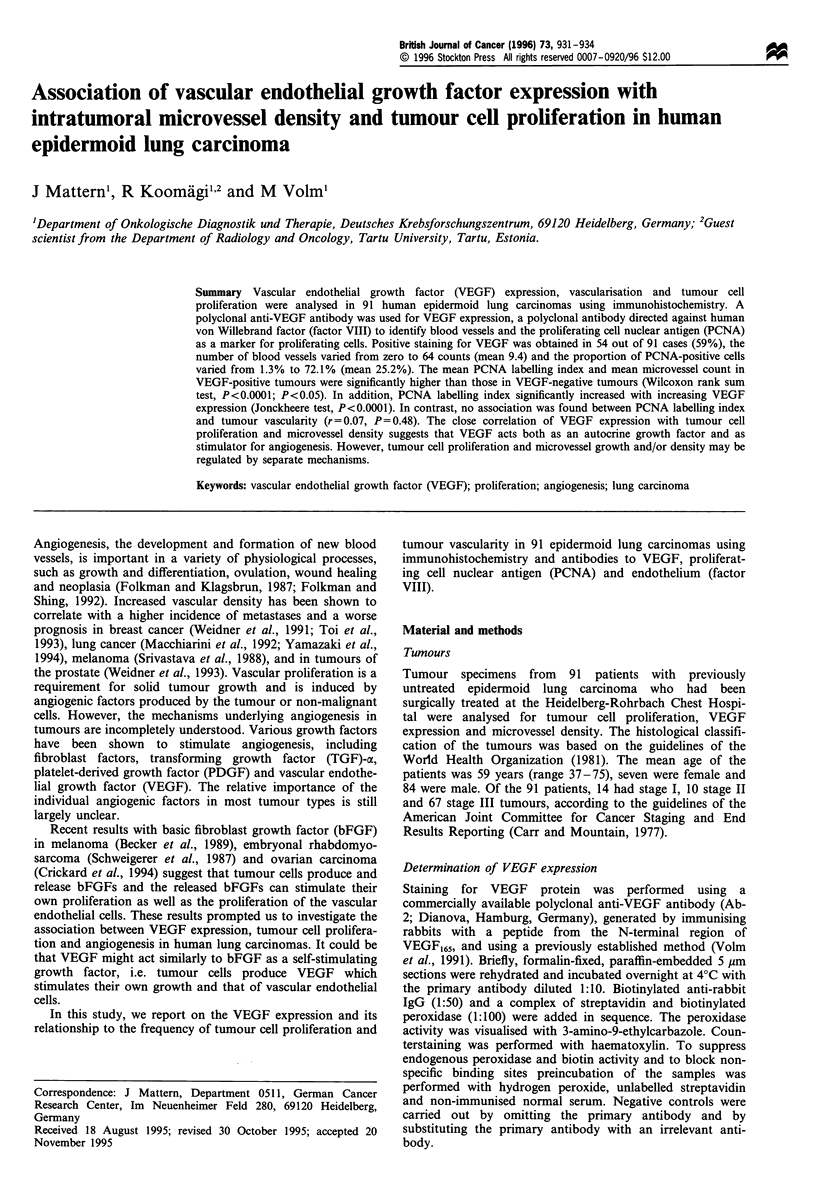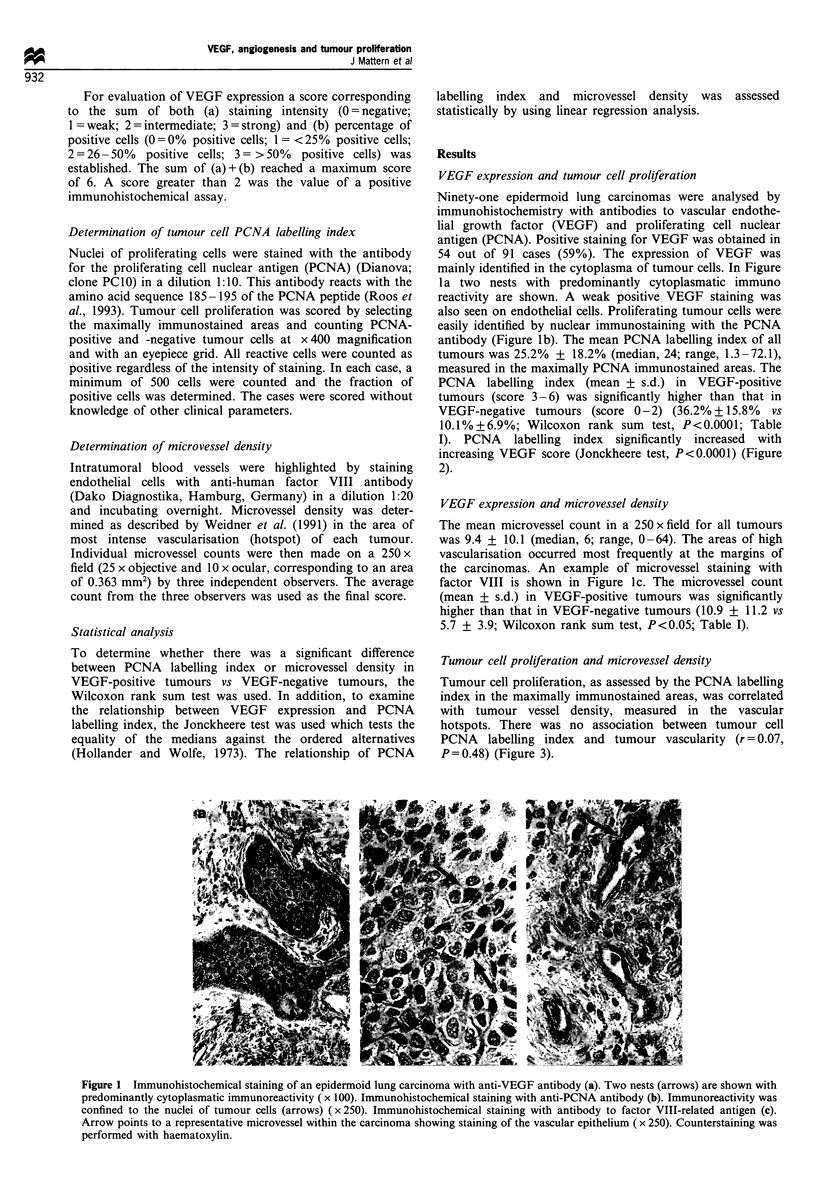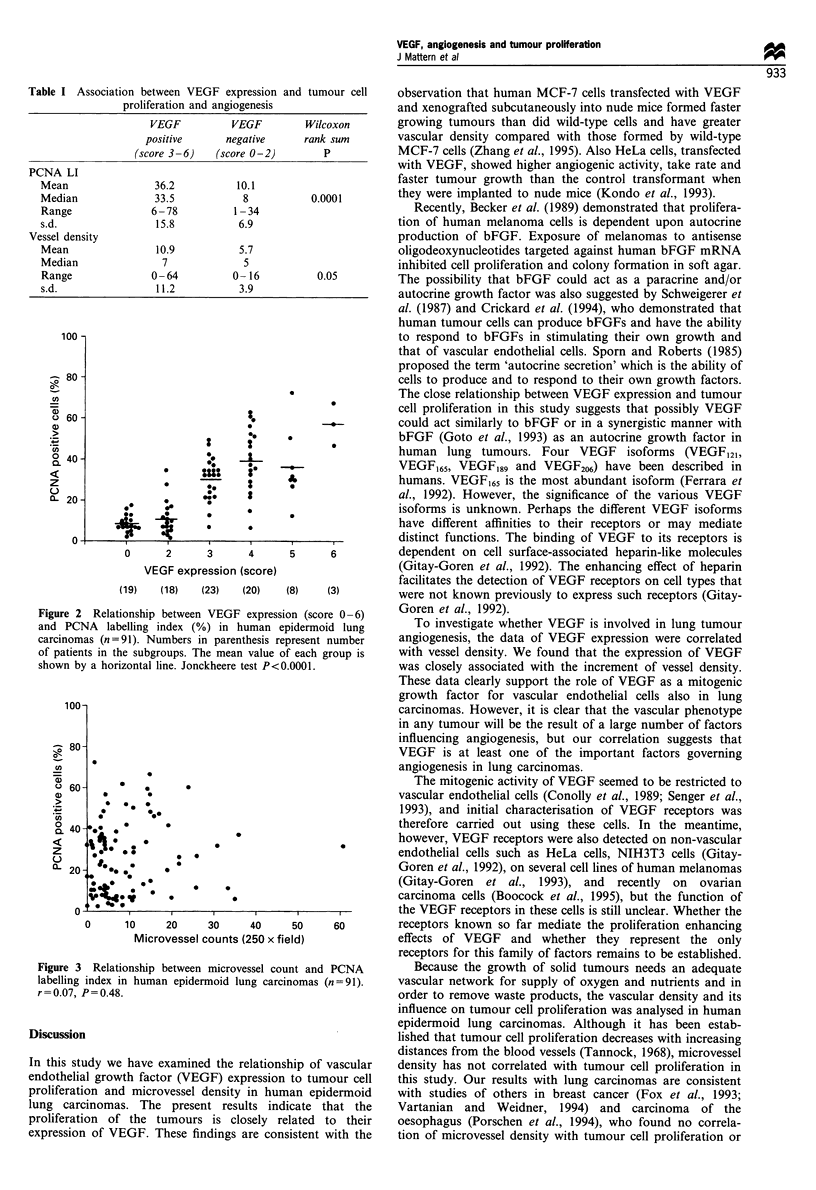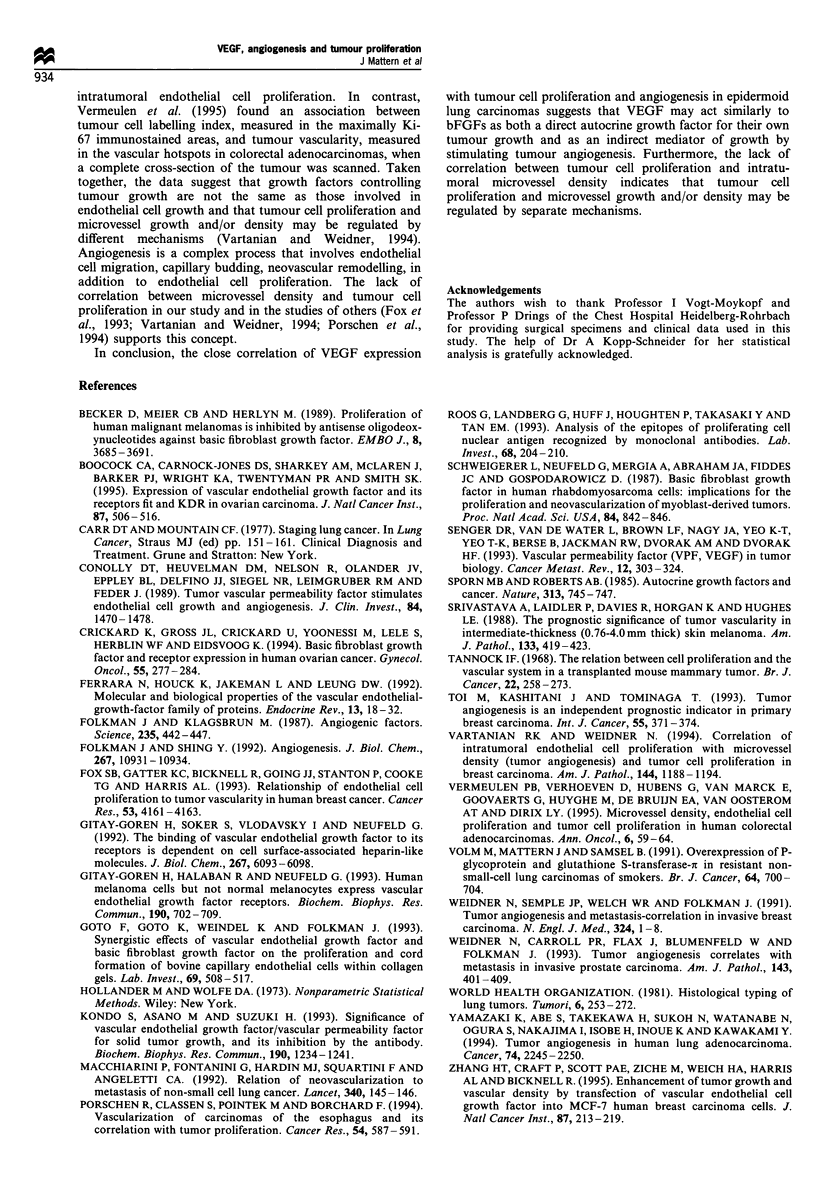# Association of vascular endothelial growth factor expression with intratumoral microvessel density and tumour cell proliferation in human epidermoid lung carcinoma.

**DOI:** 10.1038/bjc.1996.166

**Published:** 1996-04

**Authors:** J. Mattern, R. Koomägi, M. Volm

**Affiliations:** Department of Onkologische Diagnostik und Therapie, Deutsches Krebsforschungszentrum, Heidelberg, Germany.

## Abstract

**Images:**


British Journal of Cancer (1996) 73, 931-934
© 1996 Stockton Press All rights reserved 0007-0920/96 $12.00

### Association of vascular endothelial growth factor expression with intratumoral microvessel density and tumour cell proliferation in human epidermoid lung carcinoma

J Mattern[1], R Koomägi[1,2] and M Volm[1]

[1]Department of Onkologische Diagnostik und Therapie, Deutsches Krebsforschungszentrum, 69120 Heidelberg, Germany; [2]Guest scientist from the Department of Radiology and Oncology, Tartu University, Tartu, Estonia.

**Summary** Vascular endothelial growth factor (VEGF) expression, vascularisation and tumour cell proliferation were analysed in 91 human epidermoid lung carcinomas using immunohistochemistry. A polyclonal anti-VEGF antibody was used for VEGF expression, a polyclonal antibody directed against human von Willebrand factor (factor VIII) to identify blood vessels and the proliferating cell nuclear antigen (PCNA) as a marker for proliferating cells. Positive staining for VEGF was obtained in 54 out of 91 cases (59%), the number of blood vessels varied from zero to 64 counts (mean 9.4) and the proportion of PCNA-positive cells varied from 1.3% to 72.1% (mean 25.2%). The mean PCNA labelling index and mean microvessel count in VEGF-positive tumours were significantly higher than those in VEGF-negative tumours (Wilcoxon rank sum test, $P<0.0001$; $P<0.05$). In addition, PCNA labelling index significantly increased with increasing VEGF expression (Jonckheere test, $P<0.0001$). In contrast, no association was found between PCNA labelling index and tumour vascularity ($r=0.07$, $P=0.48$). The close correlation of VEGF expression with tumour cell proliferation and microvessel density suggests that VEGF acts both as an autocrine growth factor and as stimulator for angiogenesis. However, tumour cell proliferation and microvessel growth and/or density may be regulated by separate mechanisms.

**Keywords:** vascular endothelial growth factor (VEGF); proliferation; angiogenesis; lung carcinoma

Angiogenesis, the development and formation of new blood vessels, is important in a variety of physiological processes, such as growth and differentiation, ovulation, wound healing and neoplasia (Folkman and Klagsbrun, 1987; Folkman and Shing, 1992). Increased vascular density has been shown to correlate with a higher incidence of metastases and a worse prognosis in breast cancer (Weidner *et al.*, 1991; Toi *et al.*, 1993), lung cancer (Macchiarini *et al.*, 1992; Yamazaki *et al.*, 1994), melanoma (Srivastava *et al.*, 1988), and in tumours of the prostate (Weidner *et al.*, 1993). Vascular proliferation is a requirement for solid tumour growth and is induced by angiogenic factors produced by the tumour or non-malignant cells. However, the mechanisms underlying angiogenesis in tumours are incompletely understood. Various growth factors have been shown to stimulate angiogenesis, including fibroblast factors, transforming growth factor (TGF)-α, platelet-derived growth factor (PDGF) and vascular endothelial growth factor (VEGF). The relative importance of the individual angiogenic factors in most tumour types is still largely unclear.

Recent results with basic fibroblast growth factor (bFGF) in melanoma (Becker *et al.*, 1989), embryonal rhabdomyosarcoma (Schweigerer *et al.*, 1987) and ovarian carcinoma (Crickard *et al.*, 1994) suggest that tumour cells produce and release bFGFs and the released bFGFs can stimulate their own proliferation as well as the proliferation of the vascular endothelial cells. These results prompted us to investigate the association between VEGF expression, tumour cell proliferation and angiogenesis in human lung carcinomas. It could be that VEGF might act similarly to bFGF as a self-stimulating growth factor, i.e. tumour cells produce VEGF which stimulates their own growth and that of vascular endothelial cells.

In this study, we report on the VEGF expression and its relationship to the frequency of tumour cell proliferation and tumour vascularity in 91 epidermoid lung carcinomas using immunohistochemistry and antibodies to VEGF, proliferating cell nuclear antigen (PCNA) and endothelium (factor VIII).

Correspondence: J Mattern, Department 0511, German Cancer Research Center, Im Neuenheimer Feld 280, 69120 Heidelberg, Germany

Received 18 August 1995; revised 30 October 1995; accepted 20 November 1995

### Material and methods

### Tumours

Tumour specimens from 91 patients with previously untreated epidermoid lung carcinoma who had been surgically treated at the Heidelberg-Rohrbach Chest Hospital were analysed for tumour cell proliferation, VEGF expression and microvessel density. The histological classification of the tumours was based on the guidelines of the World Health Organization (1981). The mean age of the patients was 59 years (range 37-75), seven were female and 84 were male. Of the 91 patients, 14 had stage I, 10 stage II and 67 stage III tumours, according to the guidelines of the American Joint Committee for Cancer Staging and End Results Reporting (Carr and Mountain, 1977).

### Determination of VEGF expression

Staining for VEGF protein was performed using a commercially available polyclonal anti-VEGF antibody (Ab-2; Dianova, Hamburg, Germany), generated by immunising rabbits with a peptide from the N-terminal region of $VEGF_{165}$, and using a previously established method (Volm *et al.*, 1991). Briefly, formalin-fixed, paraffin-embedded 5 μm sections were rehydrated and incubated overnight at 4°C with the primary antibody diluted 1:10. Biotinylated anti-rabbit IgG (1:50) and a complex of streptavidin and biotinylated peroxidase (1:100) were added in sequence. The peroxidase activity was visualised with 3-amino-9-ethylcarbazole. Counterstaining was performed with haematoxylin. To suppress endogenous peroxidase and biotin activity and to block non-specific binding sites preincubation of the samples was performed with hydrogen peroxide, unlabelled streptavidin and non-immunised normal serum. Negative controls were carried out by omitting the primary antibody and by substituting the primary antibody with an irrelevant antibody.

For evaluation of VEGF expression a score corresponding to the sum of both (a) staining intensity (0 = negative; 1 = weak; 2 = intermediate; 3 = strong) and (b) percentage of positive cells (0 = 0% positive cells; 1 = <25% positive cells; 2 = 26–50% positive cells; 3 = >50% positive cells) was established. The sum of (a) + (b) reached a maximum score of 6. A score greater than 2 was the value of a positive immunohistochemical assay.

### *Determination of tumour cell PCNA labelling index*

Nuclei of proliferating cells were stained with the antibody for the proliferating cell nuclear antigen (PCNA) (Dianova; clone PC10) in a dilution 1:10. This antibody reacts with the amino acid sequence 185–195 of the PCNA peptide (Roos *et al.*, 1993). Tumour cell proliferation was scored by selecting the maximally immunostained areas and counting PCNA-positive and -negative tumour cells at ×400 magnification and with an eyepiece grid. All reactive cells were counted as positive regardless of the intensity of staining. In each case, a minimum of 500 cells were counted and the fraction of positive cells was determined. The cases were scored without knowledge of other clinical parameters.

### *Determination of microvessel density*

Intratumoral blood vessels were highlighted by staining endothelial cells with anti-human factor VIII antibody (Dako Diagnostika, Hamburg, Germany) in a dilution 1:20 and incubating overnight. Microvessel density was determined as described by Weidner *et al.* (1991) in the area of most intense vascularisation (hotspot) of each tumour. Individual microvessel counts were then made on a 250× field (25 × objective and 10 × ocular, corresponding to an area of 0.363 $mm^2$) by three independent observers. The average count from the three observers was used as the final score.

### *Statistical analysis*

To determine whether there was a significant difference between PCNA labelling index or microvessel density in VEGF-positive tumours *vs* VEGF-negative tumours, the Wilcoxon rank sum test was used. In addition, to examine the relationship between VEGF expression and PCNA labelling index, the Jonckheere test was used which tests the equality of the medians against the ordered alternatives (Hollander and Wolfe, 1973). The relationship of PCNA labelling index and microvessel density was assessed statistically by using linear regression analysis.

### Results

### *VEGF expression and tumour cell proliferation*

Ninety-one epidermoid lung carcinomas were analysed by immunohistochemistry with antibodies to vascular endothelial growth factor (VEGF) and proliferating cell nuclear antigen (PCNA). Positive staining for VEGF was obtained in 54 out of 91 cases (59%). The expression of VEGF was mainly identified in the cytoplasma of tumour cells. In Figure 1a two nests with predominantly cytoplasmatic immuno reactivity are shown. A weak positive VEGF staining was also seen on endothelial cells. Proliferating tumour cells were easily identified by nuclear immunostaining with the PCNA antibody (Figure 1b). The mean PCNA labelling index of all tumours was 25.2% ± 18.2% (median, 24; range, 1.3–72.1), measured in the maximally PCNA immunostained areas. The PCNA labelling index (mean ± s.d.) in VEGF-positive tumours (score 3–6) was significantly higher than that in VEGF-negative tumours (score 0–2) (36.2% ± 15.8% *vs* 10.1% ± 6.9%; Wilcoxon rank sum test, $P<0.0001$; Table I). PCNA labelling index significantly increased with increasing VEGF score (Jonckheere test, $P<0.0001$) (Figure 2).

### *VEGF expression and microvessel density*

The mean microvessel count in a 250× field for all tumours was 9.4 ± 10.1 (median, 6; range, 0–64). The areas of high vascularisation occurred most frequently at the margins of the carcinomas. An example of microvessel staining with factor VIII is shown in Figure 1c. The microvessel count (mean ± s.d.) in VEGF-positive tumours was significantly higher than that in VEGF-negative tumours (10.9 ± 11.2 *vs* 5.7 ± 3.9; Wilcoxon rank sum test, $P<0.05$; Table I).

### *Tumour cell proliferation and microvessel density*

Tumour cell proliferation, as assessed by the PCNA labelling index in the maximally immunostained areas, was correlated with tumour vessel density, measured in the vascular hotspots. There was no association between tumour cell PCNA labelling index and tumour vascularity ($r=0.07$, $P=0.48$) (Figure 3).

**Figure 1** Immunohistochemical staining of an epidermoid lung carcinoma with anti-VEGF antibody (**a**). Two nests (arrows) are shown with predominantly cytoplasmatic immunoreactivity (×100). Immunohistochemical staining with anti-PCNA antibody (**b**). Immunoreactivity was confined to the nuclei of tumour cells (arrows) (×250). Immunohistochemical staining with antibody to factor VIII-related antigen (**c**). Arrow points to a representative microvessel within the carcinoma showing staining of the vascular epithelium (×250). Counterstaining was performed with haematoxylin.

**Table I** Association between VEGF expression and tumour cell proliferation and angiogenesis

| | *VEGF positive (score 3–6)* | *VEGF negative (score 0–2)* | *Wilcoxon rank sum* P |
|---|---|---|---|
| PCNA LI | | | |
| Mean | 36.2 | 10.1 | |
| Median | 33.5 | 8 | 0.0001 |
| Range | 6–78 | 1–34 | |
| s.d. | 15.8 | 6.9 | |
| Vessel density | | | |
| Mean | 10.9 | 5.7 | |
| Median | 7 | 5 | |
| Range | 0–64 | 0–16 | 0.05 |
| s.d. | 11.2 | 3.9 | |

**Figure 2** Relationship between VEGF expression (score 0–6) and PCNA labelling index (%) in human epidermoid lung carcinomas ($n=91$). Numbers in parenthesis represent number of patients in the subgroups. The mean value of each group is shown by a horizontal line. Jonckheere test $P<0.0001$.

**Figure 3** Relationship between microvessel count and PCNA labelling index in human epidermoid lung carcinomas ($n=91$). $r=0.07$, $P=0.48$.

### Discussion

In this study we have examined the relationship of vascular endothelial growth factor (VEGF) expression to tumour cell proliferation and microvessel density in human epidermoid lung carcinomas. The present results indicate that the proliferation of the tumours is closely related to their expression of VEGF. These findings are consistent with the observation that human MCF-7 cells transfected with VEGF and xenografted subcutaneously into nude mice formed faster growing tumours than did wild-type cells and have greater vascular density compared with those formed by wild-type MCF-7 cells (Zhang *et al.*, 1995). Also HeLa cells, transfected with VEGF, showed higher angiogenic activity, take rate and faster tumour growth than the control transformant when they were implanted to nude mice (Kondo *et al.*, 1993).

Recently, Becker *et al.* (1989) demonstrated that proliferation of human melanoma cells is dependent upon autocrine production of bFGF. Exposure of melanomas to antisense oligodeoxynucleotides targeted against human bFGF mRNA inhibited cell proliferation and colony formation in soft agar. The possibility that bFGF could act as a paracrine and/or autocrine growth factor was also suggested by Schweigerer *et al.* (1987) and Crickard *et al.* (1994), who demonstrated that human tumour cells can produce bFGFs and have the ability to respond to bFGFs in stimulating their own growth and that of vascular endothelial cells. Sporn and Roberts (1985) proposed the term 'autocrine secretion' which is the ability of cells to produce and to respond to their own growth factors. The close relationship between VEGF expression and tumour cell proliferation in this study suggests that possibly VEGF could act similarly to bFGF or in a synergistic manner with bFGF (Goto *et al.*, 1993) as an autocrine growth factor in human lung tumours. Four VEGF isoforms ($VEGF_{121}$, $VEGF_{165}$, $VEGF_{189}$ and $VEGF_{206}$) have been described in humans. $VEGF_{165}$ is the most abundant isoform (Ferrara *et al.*, 1992). However, the significance of the various VEGF isoforms is unknown. Perhaps the different VEGF isoforms have different affinities to their receptors or may mediate distinct functions. The binding of VEGF to its receptors is dependent on cell surface-associated heparin-like molecules (Gitay-Goren *et al.*, 1992). The enhancing effect of heparin facilitates the detection of VEGF receptors on cell types that were not known previously to express such receptors (Gitay-Goren *et al.*, 1992).

To investigate whether VEGF is involved in lung tumour angiogenesis, the data of VEGF expression were correlated with vessel density. We found that the expression of VEGF was closely associated with the increment of vessel density. These data clearly support the role of VEGF as a mitogenic growth factor for vascular endothelial cells also in lung carcinomas. However, it is clear that the vascular phenotype in any tumour will be the result of a large number of factors influencing angiogenesis, but our correlation suggests that VEGF is at least one of the important factors governing angiogenesis in lung carcinomas.

The mitogenic activity of VEGF seemed to be restricted to vascular endothelial cells (Conolly *et al.*, 1989; Senger *et al.*, 1993), and initial characterisation of VEGF receptors was therefore carried out using these cells. In the meantime, however, VEGF receptors were also detected on non-vascular endothelial cells such as HeLa cells, NIH3T3 cells (Gitay-Goren *et al.*, 1992), on several cell lines of human melanomas (Gitay-Goren *et al.*, 1993), and recently on ovarian carcinoma cells (Boocock *et al.*, 1995), but the function of the VEGF receptors in these cells is still unclear. Whether the receptors known so far mediate the proliferation enhancing effects of VEGF and whether they represent the only receptors for this family of factors remains to be established.

Because the growth of solid tumours needs an adequate vascular network for supply of oxygen and nutrients and in order to remove waste products, the vascular density and its influence on tumour cell proliferation was analysed in human epidermoid lung carcinomas. Although it has been established that tumour cell proliferation decreases with increasing distances from the blood vessels (Tannock, 1968), microvessel density has not correlated with tumour cell proliferation in this study. Our results with lung carcinomas are consistent with studies of others in breast cancer (Fox *et al.*, 1993; Vartanian and Weidner, 1994) and carcinoma of the oesophagus (Porschen *et al.*, 1994), who found no correlation of microvessel density with tumour cell proliferation or intratumoral endothelial cell proliferation. In contrast, Vermeulen *et al.* (1995) found an association between tumour cell labelling index, measured in the maximally Ki-67 immunostained areas, and tumour vascularity, measured in the vascular hotspots in colorectal adenocarcinomas, when a complete cross-section of the tumour was scanned. Taken together, the data suggest that growth factors controlling tumour growth are not the same as those involved in endothelial cell growth and that tumour cell proliferation and microvessel growth and/or density may be regulated by different mechanisms (Vartanian and Weidner, 1994). Angiogenesis is a complex process that involves endothelial cell migration, capillary budding, neovascular remodelling, in addition to endothelial cell proliferation. The lack of correlation between microvessel density and tumour cell proliferation in our study and in the studies of others (Fox *et al.*, 1993; Vartanian and Weidner, 1994; Porschen *et al.*, 1994) supports this concept.

In conclusion, the close correlation of VEGF expression with tumour cell proliferation and angiogenesis in epidermoid lung carcinomas suggests that VEGF may act similarly to bFGFs as both a direct autocrine growth factor for their own tumour growth and as an indirect mediator of growth by stimulating tumour angiogenesis. Furthermore, the lack of correlation between tumour cell proliferation and intratumoral microvessel density indicates that tumour cell proliferation and microvessel growth and/or density may be regulated by separate mechanisms.

**Acknowledgements**
The authors wish to thank Professor I Vogt-Moykopf and Professor P Drings of the Chest Hospital Heidelberg-Rohrbach for providing surgical specimens and clinical data used in this study. The help of Dr A Kopp-Schneider for her statistical analysis is gratefully acknowledged.

### References

BECKER D, MEIER CB AND HERLYN M. (1989). Proliferation of human malignant melanomas is inhibited by antisense oligodeoxynucleotides against basic fibroblast growth factor. *EMBO J.*, **8**, 3685–3691.

BOOCOCK CA, CARNOCK-JONES DS, SHARKEY AM, McLAREN J, BARKER PJ, WRIGHT KA, TWENTYMAN PR AND SMITH SK. (1995). Expression of vascular endothelial growth factor and its receptors fit and KDR in ovarian carcinoma. *J. Natl Cancer Inst.*, **87**, 506–516.

CARR DT AND MOUNTAIN CF. (1977). Staging lung cancer. In *Lung Cancer*, Straus MJ (ed) pp. 151–161. Clinical Diagnosis and Treatment. Grune and Stratton: New York.

CONOLLY DT, HEUVELMAN DM, NELSON R, OLANDER JV, EPPLEY BL, DELFINO JJ, SIEGEL NR, LEIMGRUBER RM AND FEDER J. (1989). Tumor vascular permeability factor stimulates endothelial cell growth and angiogenesis. *J. Clin. Invest.*, **84**, 1470–1478.

CRICKARD K, GROSS JL, CRICKARD U, YOONESSI M, LELE S, HERBLIN WF AND EIDSVOOG K. (1994). Basic fibroblast growth factor and receptor expression in human ovarian cancer. *Gynecol. Oncol.*, **55**, 277–284.

FERRARA N, HOUCK K, JAKEMAN L AND LEUNG DW. (1992). Molecular and biological properties of the vascular endothelial-growth-factor family of proteins. *Endocrine Rev.*, **13**, 18–32.

FOLKMAN J AND KLAGSBRUN M. (1987). Angiogenic factors. *Science*, **235**, 442–447.

FOLKMAN J AND SHING Y. (1992). Angiogenesis. *J. Biol. Chem.*, **267**, 10931–10934.

FOX SB, GATTER KC, BICKNELL R, GOING JJ, STANTON P, COOKE TG AND HARRIS AL. (1993). Relationship of endothelial cell proliferation to tumor vascularity in human breast cancer. *Cancer Res.*, **53**, 4161–4163.

GITAY-GOREN H, SOKER S, VLODAVSKY I AND NEUFELD G. (1992). The binding of vascular endothelial growth factor to its receptors is dependent on cell surface-associated heparin-like molecules. *J. Biol. Chem.*, **267**, 6093–6098.

GITAY-GOREN H, HALABAN R AND NEUFELD G. (1993). Human melanoma cells but not normal melanocytes express vascular endothelial growth factor receptors. *Biochem. Biophys. Res. Commun.*, **190**, 702–709.

GOTO F, GOTO K, WEINDEL K AND FOLKMAN J. (1993). Synergistic effects of vascular endothelial growth factor and basic fibroblast growth factor on the proliferation and cord formation of bovine capillary endothelial cells within collagen gels. *Lab. Invest.*, **69**, 508–517.

HOLLANDER M AND WOLFE DA. (1973). *Nonparametric Statistical Methods*. Wiley: New York.

KONDO S, ASANO M AND SUZUKI H. (1993). Significance of vascular endothelial growth factor/vascular permeability factor for solid tumor growth, and its inhibition by the antibody. *Biochem. Biophys. Res. Commun.*, **190**, 1234–1241.

MACCHIARINI P, FONTANINI G, HARDIN MJ, SQUARTINI F AND ANGELETTI CA. (1992). Relation of neovascularization to metastasis of non-small cell lung cancer. *Lancet*, **340**, 145–146.

PORSCHEN R, CLASSEN S, POINTEK M AND BORCHARD F. (1994). Vascularization of carcinomas of the esophagus and its correlation with tumor proliferation. *Cancer Res.*, **54**, 587–591.

ROOS G, LANDBERG G, HUFF J, HOUGHTEN P, TAKASAKI Y AND TAN EM. (1993). Analysis of the epitopes of proliferating cell nuclear antigen recognized by monoclonal antibodies. *Lab. Invest.*, **68**, 204–210.

SCHWEIGERER L, NEUFELD G, MERGIA A, ABRAHAM JA, FIDDES JC AND GOSPODAROWICZ D. (1987). Basic fibroblast growth factor in human rhabdomyosarcoma cells: implications for the proliferation and neovascularization of myoblast-derived tumors. *Proc. Natl Acad. Sci. USA*, **84**, 842–846.

SENGER DR, VAN DE WATER L, BROWN LF, NAGY JA, YEO K-T, YEO T-K, BERSE B, JACKMAN RW, DVORAK AM AND DVORAK HF. (1993). Vascular permeability factor (VPF, VEGF) in tumor biology. *Cancer Metast. Rev.*, **12**, 303–324.

SPORN MB AND ROBERTS AB. (1985). Autocrine growth factors and cancer. *Nature*, **313**, 745–747.

SRIVASTAVA A, LAIDLER P, DAVIES R, HORGAN K AND HUGHES LE. (1988). The prognostic significance of tumor vascularity in intermediate-thickness (0.76-4.0 mm thick) skin melanoma. *Am. J. Pathol.*, **133**, 419–423.

TANNOCK IF. (1968). The relation between cell proliferation and the vascular system in a transplanted mouse mammary tumor. *Br. J. Cancer*, **22**, 258–273.

TOI M, KASHITANI J AND TOMINAGA T. (1993). Tumor angiogenesis is an independent prognostic indicator in primary breast carcinoma. *Int. J. Cancer*, **55**, 371–374.

VARTANIAN RK AND WEIDNER N. (1994). Correlation of intratumoral endothelial cell proliferation with microvessel density (tumor angiogenesis) and tumor cell proliferation in breast carcinoma. *Am. J. Pathol.*, **144**, 1188–1194.

VERMEULEN PB, VERHOEVEN D, HUBENS G, VAN MARCK E, GOOVAERTS G, HUYGHE M, DE BRUIJN EA, VAN OOSTEROM AT AND DIRIX LY. (1995). Microvessel density, endothelial cell proliferation and tumor cell proliferation in human colorectal adenocarcinomas. *Ann. Oncol.*, **6**, 59–64.

VOLM M, MATTERN J AND SAMSEL B. (1991). Overexpression of P-glycoprotein and glutathione S-transferase-$\pi$ in resistant non-small-cell lung carcinomas of smokers. *Br. J. Cancer*, **64**, 700–704.

WEIDNER N, SEMPLE JP, WELCH WR AND FOLKMAN J. (1991). Tumor angiogenesis and metastasis-correlation in invasive breast carcinoma. *N. Engl. J. Med.*, **324**, 1–8.

WEIDNER N, CARROLL PR, FLAX J, BLUMENFELD W AND FOLKMAN J. (1993). Tumor angiogenesis correlates with metastasis in invasive prostate carcinoma. *Am. J. Pathol.*, **143**, 401–409.

WORLD HEALTH ORGANIZATION. (1981). Histological typing of lung tumors. *Tumori*, **6**, 253–272.

YAMAZAKI K, ABE S, TAKEKAWA H, SUKOH N, WATANABE N, OGURA S, NAKAJIMA I, ISOBE H, INOUE K AND KAWAKAMI Y. (1994). Tumor angiogenesis in human lung adenocarcinoma. *Cancer*, **74**, 2245–2250.

ZHANG HT, CRAFT P, SCOTT PAE, ZICHE M, WEICH HA, HARRIS AL AND BICKNELL R. (1995). Enhancement of tumor growth and vascular density by transfection of vascular endothelial cell growth factor into MCF-7 human breast carcinoma cells. *J. Natl Cancer Inst.*, **87**, 213–219.